# The Prerequisites for Central Tolerance Induction against Citrullinated Proteins in the Mouse

**DOI:** 10.1371/journal.pone.0158773

**Published:** 2016-06-30

**Authors:** Robby Engelmann, Andra Biemelt, Antje Cordshagen, Anja Johl, Daniela Kuthning, Brigitte Müller-Hilke

**Affiliations:** Institute of Immunology, Rostock University Medical Center, Schillingallee 70, 18057 Rostock, Germany; University of São Paulo, BRAZIL

## Abstract

**Objectives:**

To assess the prerequisites for negative selection of peptidylcitrulline-specific T cells in the thymus. In detail, we here analyzed murine medullary thymic epithelial cells for the expression of peptidylarginine deiminases (PAD) and subsequent citrullination.

**Methods:**

Medullary thymic epithelial cells were sorted, their mRNA was isolated and the expression of *Pad* genes was analyzed by quantitative PCR. Citrullination was detected by Western Blot in lysates of sorted medullary thymic epithelial cells and histologically by immunofluorescence of thymic thin sections.

**Results:**

*Pad2* and *Pad4* are the main *Pad* isoforms expressed in mature medullary thymic epithelial cells of the mouse and their levels of expression are comparable to that of insulin (*Ins2*), another highly and promiscuously expressed protein in the thymus. Citrullination was detected in medullary thymic epithelial cells as shown by Western Blot and immunofluorescence.

**Conclusions:**

Even though we here show that the murine thymus harbors the prerequisites for central tolerance to PAD and citrullinated peptides, it remains an open question whether the emergence of peptidylcitrulline-specific T cells and of autoantibodies recognizing citrullinated epitopes is caused by a failure of central or peripheral tolerance mechanisms.

## Introduction

The presence of an autoimmune response against citrullinated epitopes is a hallmark of rheumatoid arthritis (RA). Indeed, a subset of RA patients is characterized by a distinct pattern of genetic and environmental risk factors associated with the development of antibodies against citrullinated peptide antigens (ACPA) [1]. These autoantibodies recognize citrullinated proteins that results from the posttranslational modification of arginine to citrulline. This citrullination is catalyzed by either of five isoforms of peptidylarginine deiminases (PAD) that show specific tissue distributions as well as substrate specificities [2–3]. Interestingly, while the citrullination itself is a physiological process frequently associated with inflammation [4], it is the appearance of ACPA that marks the transition to pathophysiology and RA [5].

In animal models for RA, the immunization with collagen type II in combination with complete Freund's adjuvant leads to a breakdown in tolerance, the production of ACPA and subsequent arthritis [6]. Of note, immunizing susceptible mice with citrullinated collagen was shown to induce a more severe arthritis than using native collagen and even the injection of citrullinated collagen alone, in the absence of any adjuvant, initiated arthritis [7–9]. As of yet, it is unknown why peptidylcitrulline-specific immune cells are not subjected to immune tolerance.

Immune tolerance is established at various levels during T and B cell development. As for the autoreactive T cells, central tolerance mechanisms within the thymus lead to a first wave of negative selection and elimination from the repertoire. Medullary thymic epithelial cells (mTEC) take on an important role in this process [10]. Mature mTECs express the transcriptional regulator AIRE (AutoImmune REgulator), that drives the promiscuous expression of otherwise tissue-restricted genes like e.g insulin [11–12]. During intrathymic selection, the presentation of MHC-self antigen complexes eliminates self-reactive T cells from the TCR repertoire and, by a still unclear mechanism, promotes the positive selection of self-specific regulatory T cells [13]. These regulatory T cells are essential for tolerance mechanisms in the periphery where they control autoreactive T cells that either evaded the negative selection in the thymus or recognize neo-epitopes that result from post-translational modifications [14].

We here set out to investigate whether the breach of tolerance towards citrullinated peptide antigens—as observed in RA patients and animal models of RA—can be attributed to the absence of central tolerance mechanisms. To that extent, we turned to the mouse and assessed both, the promiscuous PAD expression and citrullination in the thymus. We show that the prerequisites for central tolerance mechanisms to take effects against peptidylcitrulline-specific T cells are met.

## Methods

### Ethics Statement

All animal experiments were performed in accordance with the guidelines of the local animal use and care committee. Approval for these animal experiments by the “Landesamt für Landwirtschaft, Lebensmittelsicherheit und Fischerei Mecklenburg-Vorpommern” was not necessary as organs were taken from sacrificed mice and no experimental manipulations were performed (article 6 (1) sentence 6–9 German animal protection law). Animal housing was done by professional animal keepers and all efforts were made to minimize suffering.

### Mice

C57BL/6 (Charles River, Wilmington, MA, USA), NMRI (Charles River, Wilmington, MA, USA), DBA/1 (Charles River, Wilmington, MA, USA), MRL/MpJ (Jackson Laboratory, Bar Harbor, ME, USA) and SKG (kind gift from Ulf Hamann, DRFZ in Berlin, Germany) mice were maintained in a specific pathogen free unit on a 12 hr light / 12 hr dark cycle with 30 min twilight period. The ambient temperature was 21±2°C, the humidity was 60±10% and the room air change is 20-fold. Mice were housed using a stocking density of 3–5 mice per cage. Mice were given water and ssniff R/M-H diet (ssniff Spezialdiäten GmbH, Soest, Germany) ad libitum.

### Isolation of mature and immature mTECs

Thymic cells were isolated from 4–5 week old male mice according to a previously published workflow [15]. Mice were sacrificed by cervical dislocation prior to preparation of the thymus. Thymi from 5 mice were carefully excised and adjacent connective tissue was removed. Thymi were cut into small pieces and digested in 500 μl RPMI containing 0.02% collagenase B (Roche Deutschland Holding GmbH, Grenzach-Wyhlen, Germany), 2 U/ml dispase (BD Bioscience, San Jose, CA, USA) and 100 U/ml DNase (Roche Deutschland Holding GmbH, Grenzach-Wyhlen, Germany) per thymus. Thymic pieces were incubated at 37°C and cells were dissociated using glass pipettes with decreasing opening diameter. Single cell suspensions were centrifuged, resuspended in 4ml dense Percoll (density: 1.115 g/ml, Sigma Aldrich, St. Louis, MO, USA) and transferred into a 15 ml tube. On top of this cell suspension, 2ml of Percoll with a lower density (density: 1.065 g/ml) were layered and again on top, 2ml D-PBS (Gibco, Life Technologies, Carlsbad, CA, USA). Density gradient was established by centrifugation at 1450xg at 4°C for 30 min. TEC enriched in the second interphase were used for subsequent depletion of CD45^+^ cells using CD45-microbeads (clone: 30F11.1, Miltenyi, Bergisch Gladbach, Germany) according to manufacturer's instructions. CD45-depleted cells were stained for surface markers in ice-cold PBS pH 7.4, 0.5% bovine serum albumin, 0.1% sodium azide. The following antibodies were used: Ly-51:FITC (clone: 6C3, BD Bioscience, San Jose, CA, USA), CD80:PE (clone: 16-10A1, Biolegend, San Diego, CA, USA), CD45:PerCP (clone: 30-F11, Biolegend, San Diego, CA, USA), EpCAM:Alexa647 (clone: G8.8, Biolegend, San Diego, CA, USA). Immature mTEC (CD45^-^EpCAM^+^Ly-51^-/low^CD80^low^) and mature mTEC (CD45^-^EpCAM^+^Ly-51^-/low^CD80^high^) were sorted using a FACS Aria II machine (BD Bioscience, San Jose, CA, USA).

### PAD mRNA expression analysis

mRNA isolation was performed using the RNeasy Plus Mini Kit (Qiagen, Venlo, Netherlands) according to the manufacturer's instructions. RNA concentration and quality was checked using the Agilent RNA 6000 Pico Kit (Agilent Technologies, Santa Clara, CA, USA) again, according to the manufacturer's instructions. The mean RNA integrity number was 8.2. Reverse transcription was performed using the High Capacity cDNA Reverese Transcription kit (Applied Biosystems, Life Technologies, Carlsbad, CA, USA) according to the manufacturer's instructions. TaqMan Gene Expression Assays (Applied Biosystems, Life Technologies, Carlsbad, CA, USA) were run for following genes: *Aire* (Mm0047746_m1), *Psmb11* (Mm00613641_s1), *Ins1* (Mm01950294_s1), *Ins2* (Mm00731595_gH), *Padi1* (Mm00478062_,1), *Padi2* (Mm00447020_m1), *Padi3* (Mm00478075_m1), *Padi4* (Mm01341658_m1) and *Padi6* (Mm00462201_m1). Ct values were normalized to G*apdh* (Rodent gapdh control reagents, Applied Biosystems, Life Technologies, Carlsbad, CA, USA) by the formula 2^-Δct(gene-gapdh)^.

### Detection of citrullination by western blot

Protein lysates from FACS sorted mTECs were prepared by resuspending the cells in loading buffer (62.5 mM Tris/Hcl buffer pH 6.8 containing 2% sodium dodecyl sulfate, 65 mM dithiothreitol, 0.08% bromophenol blue and 380 mM glycerine). Lung and muscle protein lysates were prepared by traversing respective minced tissue through a 70μm cell strainer (BD Bioscience, San Jose, CA, USA) in lysis buffer (20mM Tris-HCl, pH 7.4, 10mM beta-mercaptoethanol, 100 mM NaCl, 10% glycerol). Samples were sonicated 3x for 10s each using a Laborette 19 rod sonifier at a strength of 2.2 (Fritsch GmbH, Idar-Oberstein, Germany) and passed three freeze/thaw cycles using liquid nitrogen. Thereafter, samples were centrifuged at 18,000xg at 4°C for 30 min. Supernatants were taken and diluted in loading buffer. Samples were then loaded on a Laemmli gel and SDS-PAGE was performed for 40 minutes at 220V in a miniVE Complete system (Hoefer Inc., Holliston, MA, USA). Protein was transferred to a Immobilon-FL membrane (Merck Millipore, Darmstadt, Germany) with a Trans-Blot SD Cell (Bio-Rad Laboratories GmbH, Munich, Germany). Ponceau staining was performed on the membrane to ensure sufficient protein transfer. Blocking was done over night at 4°C using Odyssey Blocking Buffer (LI-COR, Lincoln, NE, USA). Membranes were stained with 0.2μg/ml biotinylated anti-peptidyl-citrulline antibody raised against a citrullinated peptide consisting of 10 citrulline residues (clone F95, Merck Millipore, Darmstadt, Germany) or 0.1μg/ml anti-GAPDH antibody (clone 6C5, Abcam plc, Cambridge, UK) in Odyssey Blocking Buffer with 0.1% Tween-20 for 2 hours at room temperature. After washing Streptavidin-DyLight800 (Thermo Fisher Scientific, Waltham, MA, USA) or anti-mouse-IRDye680CW (LI-COR, Lincoln, NE, USA) were incubated in Odyssey Blocking Buffer with 0.1% Tween-20 for 1h to visualize citrulline or GAPDH staining, respectively. Membranes were scanned and bands quantified with a Odyssey CLx Scanner (LI-COR, Lincoln, NE, USA).

### Immunofluorescence detection of citrullination

Thymi were excised, mounted onto a steel block by Tissuetek (Sakura Finetek, Alphen an den Rijn, Netherlands) and frozen in liquid nitrogen. 6 μm cryosections were prepared using a Carl Zeiss cryotome (Carl Zeiss Jena GmbH, Jena, Germany) and were mounted on glass slides. The sections were fixed for 10 min in -20°C pre-cooled acetone (LI-COR, Lincoln, NE, USA), and were then dried for 20 min at RT. They were washed two times for 5 min each with TBS-buffer. The sections were encircled with a wax pen (Thermo Fisher Scientific Inc., Waltham, MA, USA) and were blocked with blocking buffer (1x TBS containing 2.5% skim milk (USBiological, Salem, MA, USA) and 2.5% FCS (Biochrom GmbH, Berlin, Germany) for 1 h in a humidified chamber at RT. Primary reagents in the respective dilutions were added to the sections and incubated overnight in a humidified chamber at 4°C. Following primary reagents were used: rat-anti-mouse Aire IgG2 (clone 5H12; eBioscience San Diego, CA, USA; dilution: 1:100), rabbit anti-Cytokeratin 5 antibody (Covance, Princeton, NJ, USA; dilution: 1:1000), rabbit anti-citrullin (Abcam, Cambridge, UK; dilution: 1:500), Rabbit Serum (dilution 1:100), human ACPA-positive sera pool (dilution: 1:100), human ACPA-negative sera pool (dilution: 1:100) and UEA-1 lectin coupled to biotin (Vector Laboratories, Burlingame, CA, USA; dilution: 1:100). The sections were washed two times for 5 min each with TBS-buffer and the respective dilutions of the secondary reagents were added. Subsequently, the sections were incubated for 1 h at RT in a humidified chamber. As secondary reagents, the following antibodies were used: goat-anti-rat IgG Alexa488 (Molecular Probes, Life Technologies, Carlsbad, CA, USA; dilution: 1:500), goat-anti-rabbit IgG Alexa488 (Molecular Probes, Life Technologies, Carlsbad, CA, USA; dilution: 1:500), goat-anti-rabbit IgG Alexa546 (Molecular Probes, Life Technologies, Carlsbad, CA, USA; dilution: 1:500), goat-anti-human IgG Alexa488 (Molecular Probes, Life Technologies, Carlsbad, CA, USA; dilution: 1:500) and Streptavidin Alexa546 (Molecular Probes, Life Technologies, Carlsbad, CA, USA; dilution: 1:500). The sections were washed two times for 5 min each with TBS-buffer. DAPI (Molecular Probes, Life Technologies, Carlsbad, CA, USA; 1:1000 dilution) was added onto the sections and incubated for 15 min at room temperature. The sections were again washed two times for 5 min each with TBS-buffer, four times for 30 s each under running tap water and one time for 5 min with distilled water. The excess water was removed carefully from the slides and the sections were embedded in Fluorescence Mounting Medium (DiaSorin Inc., Stillwater, MN, USA). Confocal images were acquired using the LSM 780 (Carl Zeiss Jena GmbH, Jena, Germany) using the software ZEN2011.

### Statistics

Statistical analyzes were performed in R (Version 3.1.2). Differences between two groups were tested by Mann-Whitney U-test or student’s T-test where appropriate. Correlations were calculated by Spearman's rank correlation. Confidence intervals were calculated by spearman.ci function (R-package: RVAideMemoire).

## Results

### Sorting of mature and immature mTEC

**Fig 1:**
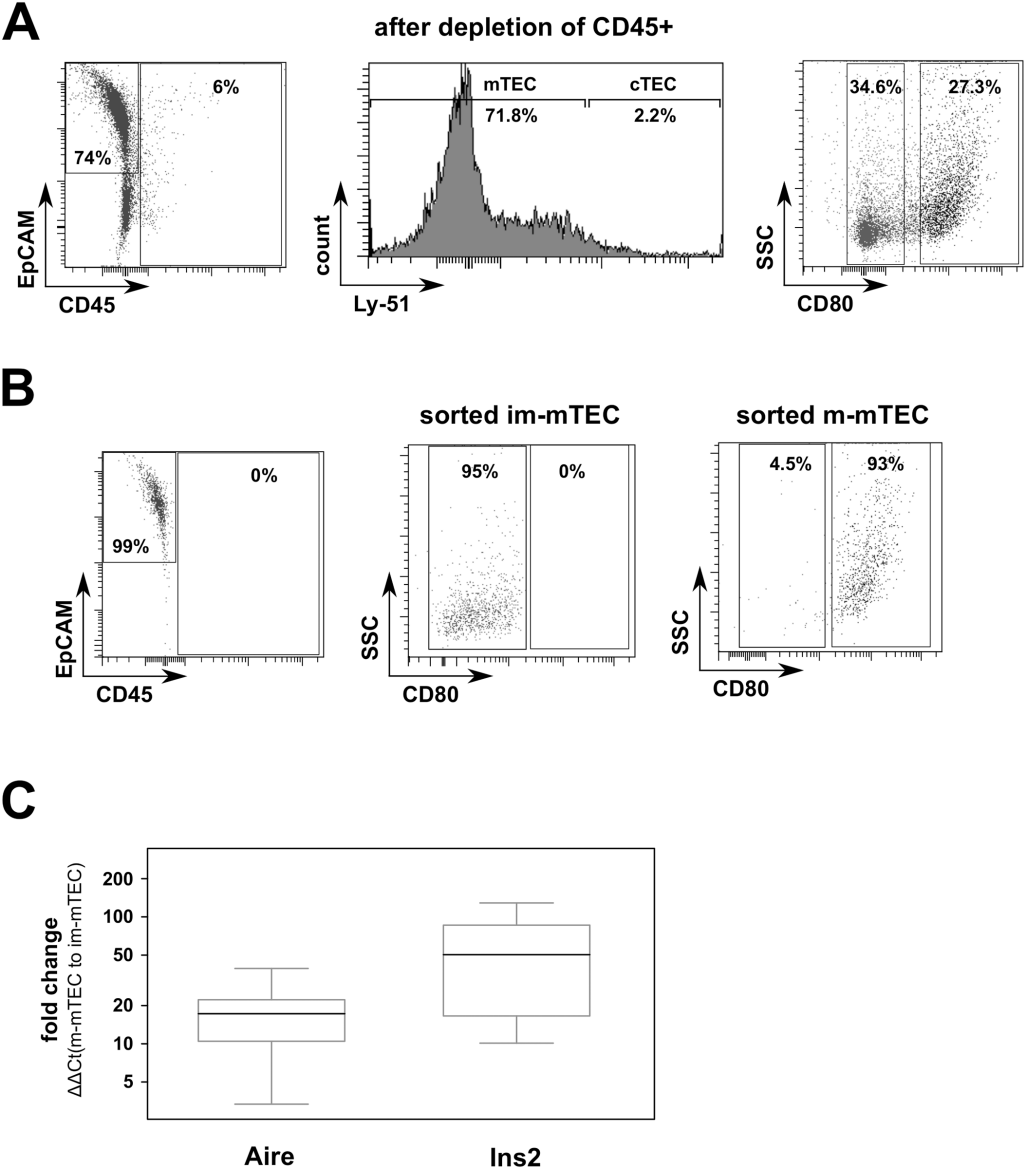
Isolation of mature and immature mTECs from murine thymi. (A) Flow cytometric analyses of thymic cells from NMRI mice, magnetically depleted of CD45-positive cells. TECs are identified as EpCAM-positive and CD45-negative cells (left panel). mTECs were considered as Ly-51^-/low^ cells (middle panel). According to their CD80-expression, we further discriminated mature mTECs (m-mTEC) and immature mTECs (im-mTEC) (right panel). (B) Re-analyses showed high purities of m-mTEC (right panel) and im-mTEC (middle panel). (C) mRNA expression of *Aire* and *Ins2* was higher in mature compared to immature mTEC. *Gapdh* was used as the reference gene. Boxes show medians and quartiles. Data shown are pooled from C57BL/6, DBA/1, MRL/MpJ and SKG mice.

In order to investigate a promiscuous PAD expression in the medulla of the murine thymus, we first isolated the mTECs via FACS. To that extent we identified all TECs as CD45-negative and EpCAM-positive cells and then differentiated the Ly-51^hi^ cTECs from the Ly-51^lo/negative^ mTECs. Mature and immature mTECs were further differentiated on the basis of their CD80 expression (Fig 1A). We isolated mature and immature mTECs with mean purities of 86.9% and 88.5%, respectively (Fig 1B). As leukocytes were previously shown to express PAD isoforms [2], we thoroughly controlled our sorted cells for impurities. Indeed, the mean percentages of CD45-positive leukocytes among the sorted mature and immature mTECs were 2.1% and 1.6% only and thus negligible. The mean percentages of Ly-51^hi^ cTECs among sorted immature and mature mTECs were 3.6% and 0.1%, respectively. To further confirm the identity of both mTEC populations, we analyzed the expression of *Aire* and *Ins2* as examples for an AIRE-dependent promiscous gene expression [16]. To that extent, we isolated the mRNA from the sorted cell populations and performed qRT-PCR for *Gapdh* as well as our genes of interest. We thus found a median 17-fold (IQR: 11.8; p<0.001) increased expression for *Aire* in mature compared to immature mTECs. Likewise, there was a 50-fold (IQR: 69.5; p<0.001) higher expression for *Ins2* in mature mTECs compared to immature mTECs (Fig 1C).

### PAD genes are readily expressed in mTEC

**Fig 2:**
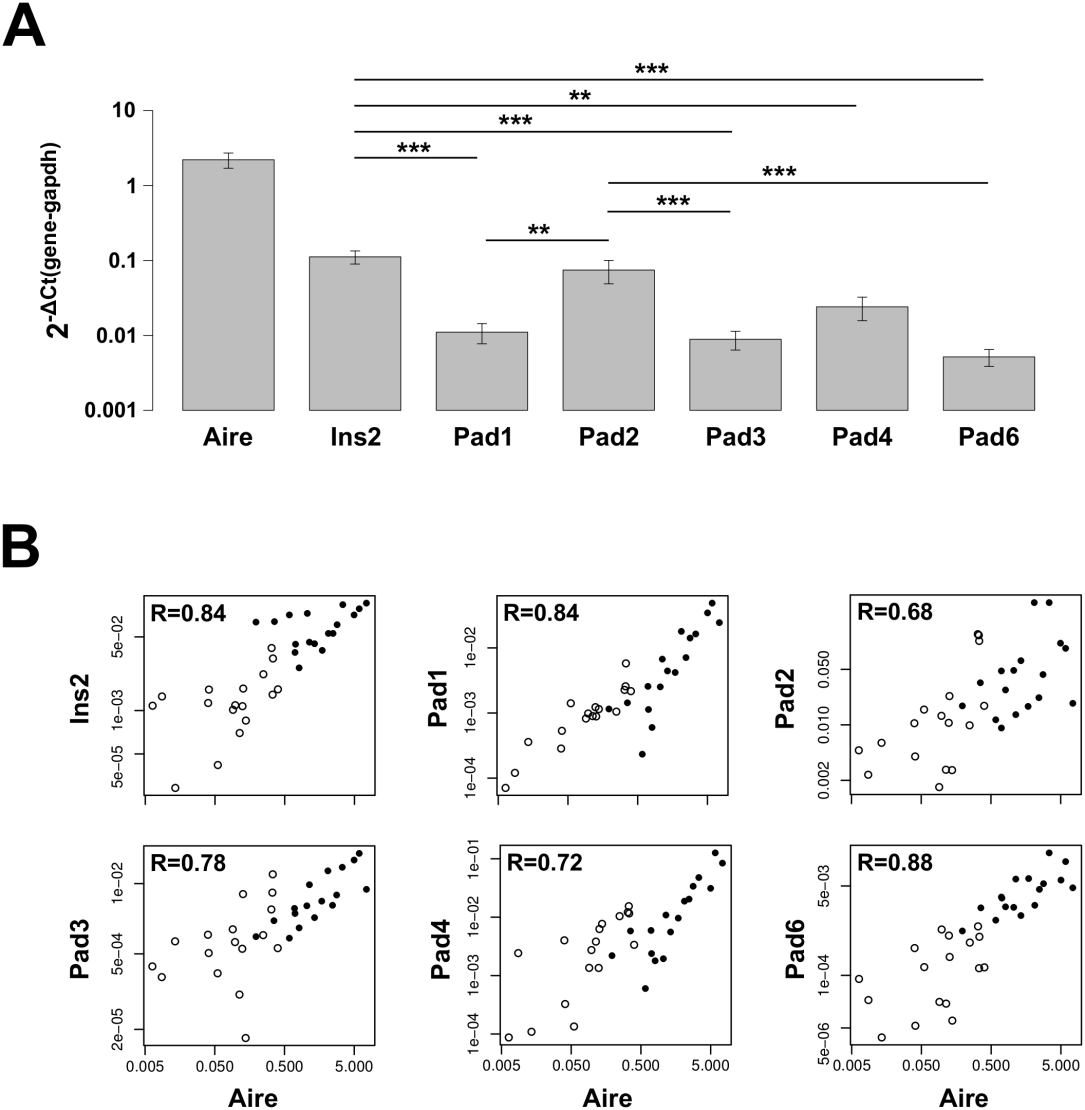
*Pad*-RNA is expressed in mTECs. (A) mRNA expression of *Aire*, the tissue-restricted gene *Ins2* as well as five *Pad* isoforms was analyzed in mature mTECs performing qPCR. *Gapdh* was used as a reference gene. Bars show mean +/- SEM (n = 20). p-values were calculated by t-test. (B) *Aire* expression is correlated to the expression of *Ins2* and all five *Pad* isoforms. Open and closed circles indicate the expression in immature and mature mTEC, respectively. The correlation coefficients were calculated by Spearman test. All p-values were below 0.001. The results shown are pooled data derived from experiments performed with C57BL/6, DBA/1, MRL/MpJ and SKG mice.

We next used the isolated mTECs to analyze the expression of the five *Pad* isoforms at the mRNA level. We could show, that out of the five *Pad* isoforms, four are significantly higher expressed in mature mTECs compared to immature mTECs (*Pad1*: 0.01 vs 0.001, p = 0.003; *Pad3*: 0.008 vs. 0.002, p = 0.02; *Pad4*: 0.02 vs. 0.005, p = 0.03; *Pad6*: 0.005 vs. 0.0003, p<0.001), whereas *Pad2* showed no significant difference in the expression level between mature and immature mTECs (*Pad2*: 0.07 vs. 0.03, p = 0.13) (*Pad* expression in mature and immature mTECs is shown in Fig 2B). Interestingly, *Pad2* showed the highest expression level in mature mTECs and was comparable to *Ins2*. In contrast, the other four *Pad* isoforms showed a significantly lower expression than *Ins2* and—except for *Pad4 –*also a significant lower expression than *Pad2* (Fig 2A). Therefore, we conclude that *Pad2* and *Pad4* are the main *Pad* isoforms expressed in mature mTECs in mice and their expression levels are comparable to *Ins2*. The comparison of *Pad* expression among various mouse strains—some of which are prone to the development of autoimmunity—showed no significant differences.

Of note, we did find significant correlations between the expression of *Aire* and *Ins2* (R = 0.84, CI 0.71–0.91), as well as between *Aire* and all five *Pad* isoforms (*Pad1*: R = 0.85, CI 0.63–0.95; *Pad2*: R = 0.68, CI 0.43–0.82; *Pad3*: R = 0.78, CI 0.59–0.89; *Pad4*: R = 0.72, CI 0.44–0.88 and *Pad6*: R = 0.88, CI 0.76–0.93) (Fig 2B).

### Citrullination is present in mTECs and in the medulla of the thymus

**Fig 3:**
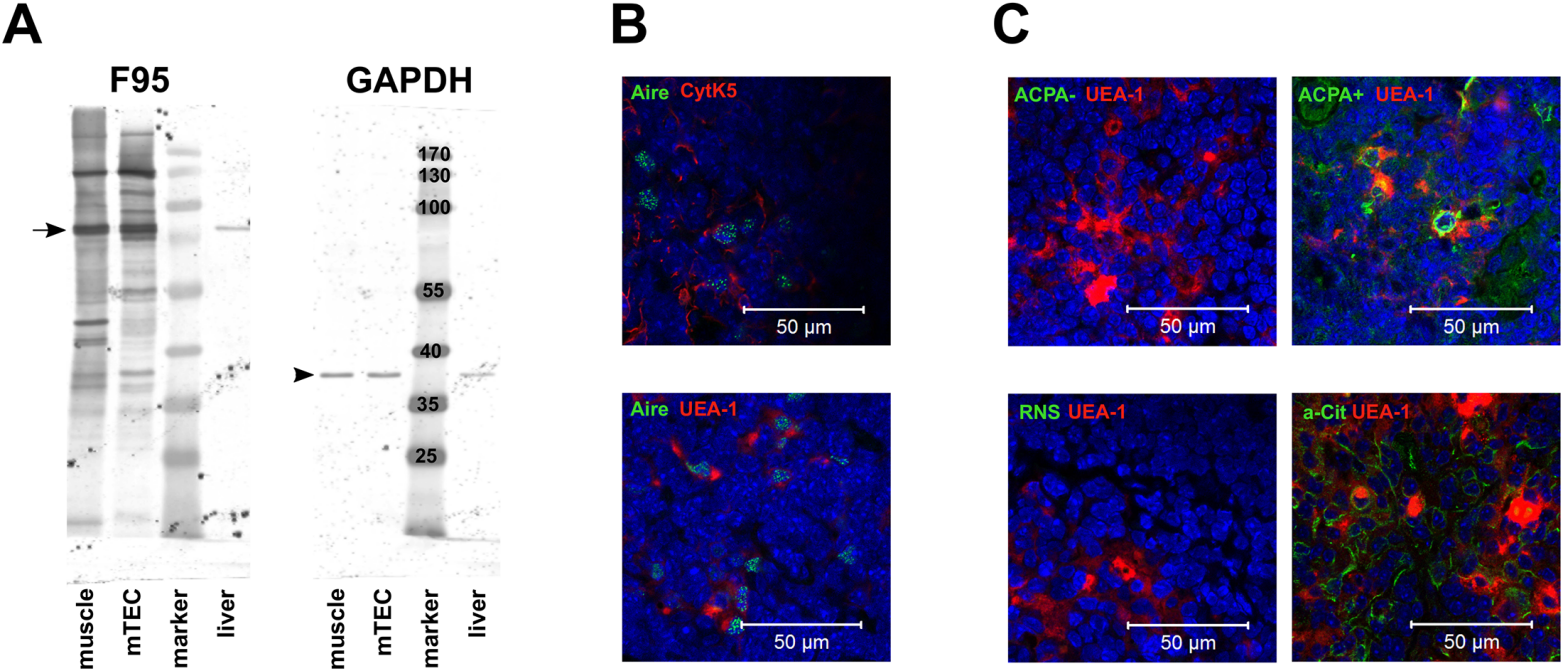
Citrullination is present in isolated mTEC and within medullary areas of the thymus. (A) Western blot to detect citrullination (clone F95) and the reference protein (GAPDH) in lysates from FACS sorted mTECs, muscle (positive control) and liver (negative control). The arrow indicates the band present in all samples that was used for a relative expression analysis with respect to GAPDH (arrowhead): muscle: 21-fold, mTEC: 28-fold and liver: 0.75-fold. Numbers within the band of the protein marker lane indicate protein sizes in kDa. (B) Identification of mTEC in thin sections of murine thymi by dual immunofluorescence staining with anti-Aire (green) and anti-cytokeratin 5 (red) or UEA-1 (red). (C) Detection of citrullination in mTEC areas of thymic thin sections. mTECs were stained with UEA-1 (red) and RA-specific epitopes are labeled with an ACPA-high serum mixture consisting of three RA patient's sera (green) (upper right panel). A mix of two age-matched healthy control sera was used as negative control (upper left panel). Staining with a rabbit anti-citrulline serum (green) shows citrullination within mTEC areas (lower right panel). Rabbit normal serum was used as a negative control (lower left panel). All results shown were derived from NMRI thymi.

The appearance of *Pad* mRNA in murine mTECs prompted us to investigate whether citrullination does take place in these cells. Therefore, we took two independent approaches. Firstly, we isolated protein from FACS sorted mTECs and performed a western blot including muscle tissue and liver lysate as positive and negative controls, respectively. As shown in Fig 3A we clearly could detect multiple bands stained with the F95 anti-citrulline antibody in the lysates of mTECs and muscle. As expected, liver lysates completely lacked the staining for citrullination except for a single weak band at around 90 kDa. We analyzed the relative expression of this band in reference to the GAPDH expression in all three lysates and found the highest relative expression in mTECs (28-fold), followed by muscle (21-fold) and liver (0.75-fold).

Secondly, we performed immunofluorescence on thymic thin sections. We identified mTECs in these thin sections of mouse thymi by staining with antibodies against cytokeratin 5 and AIRE as well as with the lectin UEA-1 (Fig 3B). The detection of citrullination was performed two-fold: First, we mixed sera of three ACPA-high RA-patients and used the mix of two age and sex-matched healthy control sera as a negative control. While there was no staining with the negative control sera, we could show a clear staining with the ACPA-positive sera within mTEC areas of murine thymi (Fig 3C). Thus, epitopes recognized by RA-specific antibodies exist within the thymic medulla. In a next step, we directly showed citrullination by using an anti-citrulline antibody. Indeed, we could demonstrate that citrullinated proteins are present within the mTEC areas of mouse thymi (Fig 3C). These results hold true for all mouse strains investigated and do not reveal any strain-specific differences.

## Discussion

We here demonstrate the expression of *Pad* isoforms in murine mTECs. We also show that *Pad2* is expressed at a comparable level with *Ins2* and *Pad4* and we therefore conclude that *Pad2* and *Pad4* are the main *Pad* isoforms expressed in murine mTECs. Interestingly, PAD2 and PAD4 have also been shown to be upregulated in monocytes and macrophages of the RA synovium and that they contribute to the citrullination of synovial proteins [17]. It can therefore be anticipated, that the very same epitopes become citrullinated in mature mTEC as well as in the RA synovium.

We further analyzed whether citrullination—the result from PAD protein expression and PAD activity—does take place in mTECs. Indeed, performing Western Blot analyses, we demonstrated that murine mTECs express citrullinated proteins at a level comparable to muscle tissue. Of note, lysates from murine muscle tissue but not from liver were previously shown to exhibit a high PAD activity [18]. As a second independent method, we performed immunofluorescence on thymic thin sections and we here show that citrullinated epitopes are present in medullary areas. These citrullinated epitopes are recognized by both, the sera of ACPA-positive RA patients and a peptidylcitrulline-specific antibody, whereas sera of age matched ACPA-negative controls and rabbit normal serum did not bind. In summary, our data indicate that the prerequisites for a negative selection of peptidylcitrulline-specific T cells in the thymus are met. However, because we did not investigate negative selection itself, we cannot predict whether peptidylcitrulline-specific T cells manage to escape into the periphery. Moreover, it needs to be determined whether thymocytes can be induced to differentiate into peptidylcitrulline-specific regulatory T cells. It therefore remains an open question whether autoimmunity in RA is driven by a failure of central or peripheral tolerance mechanisms [19–20].

*Ins2* has previously been shown to be expressed in an AIRE-dependent manner [21]. In line with this, we found a correlation between the expression levels of *Ins2* and *Aire*. In addition, we found correlations between *Aire* expression and the expression of *Pad* isoforms. This is suggestive of *Pad* genes also being regulated by the transcriptional regulator AIRE, even though we do not yet have a formal proof for a direct regulation. Interestingly, a recent genetic association study in human RA patients linked an allelic variation in the fifth exon of *AIRE* to an increased susceptibility for RA. This variation is associated with a lower expression of AIRE [22]. One may therefore speculate, that a lower AIRE expression in individuals carrying the variation can also lead to a decreased expression of PAD isoforms, and subsequently to a reduced citrullination in mTECs which in turn can result in an increased frequency of peptidylcitrulline-specific T cells escaping central tolerance.

In summary, we here conclude that the prerequisites for a negative selection of peptidylcitrulline-specific T cells are met in the murine thymus. Ongoing experiments are underway to address whether this holds true for the human situation and whether T cells are actually negatively selected against citrullinated epitopes.

## Supporting Information

S1 File TaqMan data summary. Delta Ct values expressed as 2^(-deltaCt(gene-gapdh)) grouped for mature and immature mTECs as well as for mouse strain.
(CSV)
